# Multi-omics analyses of the mechanism for the formation of soy sauce-like and soybean flavor in *Bacillus subtilis* BJ3-2

**DOI:** 10.1186/s12866-022-02555-5

**Published:** 2022-05-20

**Authors:** Yongjun Wu, Yi Tao, Jing Jin, Shuoqiu Tong, Sheng Li, Lincheng Zhang

**Affiliations:** Key Laboratory of Plant Resource Conservation and Germplasm Innovation in Mountainous Region (Ministry of Education), Collaborative Innovation Center for Mountain Ecology & Agro-Bioengineering (CICMEAB), College of Life Sciences/Institute of Agro-Bioengineeringering, Guizhou University, Guiyang, 550025 Guizhou Province China

**Keywords:** Soy sauce-like flavor, Soybean flavor, Bacillus subtilis, Transcriptome, Proteomics, Metabolomics, Multi-omics

## Abstract

Although soy sauce-like flavor and soybean flavor are two key contributors to the flavor of fermented foods, the key compounds of soy sauce-like flavor and soybean flavor and production mechanisms are still poorly understood and need further investigation. In the present study, we found that the *Bacillus subtilis* (*B. subtilis*) BJ3-2 strain has various metabolic properties at different temperatures, and the strain cultured at 37℃ increased the soybean flavor (a special flavor of ammonia-containing smelly distinct from natto) compared with culturing at 45℃ and 53℃. Interestingly, the strain cultured at 45℃ and 53℃ had a higher soy sauce-like flavor than that in 37℃. Moreover, a comparative transcriptome analysis of the strain cultured at 37℃, 45℃, and 53℃ showed transcriptional changes related to secondary metabolites and ABC transporters, which is critical for the amino acid transport and metabolism in *B. subtilis*. Meanwhile, proteomics and metabolomics profiling showed a marked change in amino acids transport and metabolism. In addition, the metabolic analysis revealed a significant metabolic difference (including sulfur metabolism, glutathione metabolism, nicotinate and nicotinamide metabolism, cysteine and methionine metabolism, and pyrimidine metabolism) in the strain cultured at 45℃ and 53℃ compared to 37℃. To sum, this study used the multi-omics profiling tool to investigate the fermentative strains *B. subtilis* BJ3-2, thus providing a deeper insight into the mechanism of the formation of soy sauce-like flavor and soybean flavor compounds.

**Supplementary Information:**

The online version contains supplementary material available at 10.1186/s12866-022-02555-5.

## Introduction

The unique flavor and nutritional value of the fermented foods result from numerous microorganisms and their metabolites produced during fermentation [1, 2]. The soy sauce-like flavor is one of the most popular flavors among consumers worldwide, attributed to its appealing flavors and beneficial health effects in fermented beverages [3, 4]. *B. subtilis* is one of the most dominant microorganisms found in fermented foods [5, 6]. Fermentation by *B. subtilis* is an inexpensive and effective process that improves nourishing and functional food ingredients [7, 8]. For instance, *B. subtilis* can produce a variety of components during fermented processes, such as protease, amylase, and lipase, all of which have important roles in the hydrolysis of proteins, starches, and lipids [9]. In addition, the chemicals produced by the carbon metabolism of *B. subtilis* significantly contribute to the formation of flavor compounds during the Baijiu brewing process [10]. Therefore, *B. subtilis* has been acknowledged as an important bacterium for forming flavor compounds in fermented foods [5, 9, 11].

Among all the flavor chemicals, acetoin and ligustrazine, produced by *B. subtilis* have been widely reported as greatly impacting the flavor of fermented foods and other foods [12–15]. Tetramethylpyrazine (TTMP) is a commonly occurring alkylpyrazine that has been considered an important contributor to aroma compounds in oriental foods, such as lobster sauce (a Chinese fermented soybean product produced by *B. subtilis*). Homoplastically, natto is a traditional Japanese food obtained after fermentation of the soybeans by *B. subtilis* [16, 17]. However, these traditional foods' main compositions and key flavor compounds also tend to differ. In addition, TTMP has also been identified as one of the main contributors to the *Maotai-flavor* of Chinese liquors [18]. Moreover, several alkylpyrazines were produced by *B. subtilis* grown in solid substrate conditions using soybean suspended in water and supplemented with threonine and acetoin [19] as precursors of TTMP. Acetoin (3-hydroxy-2-butanone or acetyl methyl carbinol), a very active molecule, is a precursor of dozens of compounds. Previous studies have extensively researched the biological synthesis pathways and nonenzymatic spontaneous reactions of acetoin and its derivatives [20, 21]. It has also been reported that acetoin is a precursor of TTMP that can synthesize MP via microbial metabolism during Baijiu fermentation [15, 22]. Moreover, acetoin and TTMP have recently drawn extensive attention due to their flavor-promoting role [23, 24]. Yet, the key flavor compositions related to the soy sauce-like aroma style were complex and with low content in fermented foods [25].

Recently, a dominant strain of *Bacillus licheniformis* isolated from the Maotai flavor liquor produced a strong soy sauce-like flavor in the medium of wheat bran extract by submerged fermentation [26]. More importantly, cysteine was identified as having an important role in forming soy sauce-like flavor compounds. It was also suggested that it might act as an indirect precursor or stimulator of soy sauce-like flavor formation [26]. Additionally, it has also been reported that sulfur-containing aroma compounds formed from nonvolatile cysteinylated precursors are key contributors to the flavor of a diverse range of foods and beverages [27]. Moreover, approximately 76 volatile compounds have been well identified and characterized in Chinese soy sauce-like aroma style liquor, such as esters, alcohols, aldehydes and ketones, and aromatic compounds [28]; however, most of them did not exhibit soy sauce-like flavor. The indetermination related to the key soy sauce-like aroma compositions led to confusion about the formation mechanism of the flavor compounds. Therefore, more information and further studies about the key flavor compositions related to the soy sauce-like aroma style are needed.

In our previous study, a strain of *B. subtilis* BJ3-2 were isolated and identified from the bacteria-fermented lobster sauce samples which collected from 10 producing areas in Guizhou province [29]. In this study, we discovered that BJ3-2 has various metabolic properties at different temperatures. E.g., the BJ3-2 strain cultured at 37℃ increased the soybean flavor compared with culturing at 45℃ and 53℃. On the contrary, the strain cultured at 45℃ and 53℃ had a higher soy sauce-like flavor than that cultured at 37℃. Furthermore, the genes involved in physiological metabolism at the molecular level were analyzed by a comparative transcriptome. Additionally, the proteins and metabolites involved in physiological metabolism were detected by proteomics and metabolomics. The present study results provide a scientific basis for the information on the ingredients of key soy sauce-like flavor and soybean flavor compounds. They can also be beneficial for the development of the fermentative industry.

## Materials and methods

### The culture and collection of Bacillus subtilis BJ3-2

The culture and collection of *Bacillus subtilis* BJ3-2 as previously described [29, 30]. Briefly, the BJ3-2 strains were cultured overnight in solid LB (Luria–Bertani) at 37 °C, after which a single colony on the plate was picked and cultured again under previously described conditions. These procedures were repeated at least three times for activation of the colony. Thereafter, a single activated colony on the plate was picked and grown in 5 mL liquid LB medium at 37 °C for 5 h under shaking at 180 rpm. The above bacterial cultures (1 mL) were reinoculated in 99 mL of liquid LB medium (1%) and grown at 37 °C under shaking at 180 rpm until an OD600 of 0.70. The 3 mL above cultures were then reinoculated in 297 mL liquid LB medium and grown at 37 °C, 45 °C, and 53 °C (shaking at 180 rpm, 42 h) for the enlarged cultivation process. Then, the enlarged cultures of each sample were transferred to a new 50 mL tube, and the precipitate was collected after being centrifuged at 12,000 rpm for 10 min. For RNA sequencing, the precipitate of each sample cultured at 37℃, 45℃, and 53℃ was named as AT37, BT45, and CT53, respectively. For the proteomic analysis, the precipitate of each sample cultured at 37℃, 45℃, and 53℃ was named AP37, BP45, and CP53, respectively. For the metabolomics analysis, the precipitate of each sample cultured at 37℃, 45℃, and 53℃ was named as A37, B45, and C53, respectively. Each treatment was performed at least in triplicate for all samples. The sensory analysis of the strain cultured at 37℃, 45℃, and 53℃ was evaluated by trained expert sensory panelists.

### RNA sequencing

The total RNA was extracted using TRIzol reagent (Invitrogen, Carlsbad, CA, USA) by following the manufacturer's protocol. The concentration and purity of the total RNA were evaluated using a Nanodrop ND-2000 spectrophotometer (Thermo Fisher Scientific, Wilmington, DE, USA). The RNA integrity values of the total RNA were determined using an Agilent 2100 bioanalyzer (Agilent, Palo Alto, CA, USA). Each sample was collected at least three times and pooled for RNA sequencing (RNA-seq) analysis. RNA-seq was performed by the Majorbio Technology Co., Ltd. (Shanghai, China). The 16S rRNA was used as an internal standard. Sequences of the raw data containing a small number of reads with sequencing adaptors or low-quality sequences were filtered out. Subsequently, the quality of sequencing data was evaluated by a sequencing error rate distribution examination. For gene expression analysis, the reads counts were normalized to Fragments Per Kilo bases per Million fragments (FPKM), and the differentially expressed genes (DEGs) were selected using the following conditions: *P*-value < 0.05 and |log_2_fold change (FC) |≥ 1. Gene Ontology (GO, http://www.geneontology.org/) enrichment analysis was performed to determine the DEGs that were common at 37 °C, 45 °C, and 53 °C with opposite regulatory patterns with a threshold of P < 0.05. Kyoto Encyclopedia of Genes and Genomes (KEGG, http://www.genome.jp/kegg/) pathway enrichment analysis was performed for each DEG [31].

### nanoLC-MS/MS analysis

For each sample, 2 μg of total peptides were separated and analyzed with a nano-UPLC (EASY-nLC1200) coupled to a Q Exactive HFX Orbitrap instrument (Thermo Fisher Scientific) with a nano-electrospray ion source according to the method of Liu with some modifications [32]. Separation was performed using a reversed-phase column (100 µm ID × 15 cm, Reprosil-Pur 120 C18-AQ, 1.9 µm, Dr. Maisch). Mobile phases were H_2_O with 0.1% FA, 2% ACN (phase A) and 80% ACN, 0.1% FA (phase B). Separation of the sample was performed with a 90 min gradient at 300 nL/min flow rate; Gradient B: 2–5% for 2 min, 5–22% for 68 min, 22–45% for 16 min, 45–95% for 2 min, 95% for 2 min. The data acquisition was performed with the data-dependent acquisition (DDA) in profile and the DDA of MS1 spectra was performed in positive mode with an Orbitrap analyzer at a resolution of 120,000 (at m/z of 200) and 350–1600 m/z range. The MS2, resolution was set at 45 k and fixed first mass was set to 100 m/z. The automatic gain control target was set to 3E6 (with the max IT 30 ms) and 1E5 (with the max IT 96 ms) in MS1 and MS2, respectively. The top 20 most intense ions were fragmented by high-energy dissociation (HCD) with normalized collision energy of 32% and isolation window of 0.7 m/z. The dynamic exclusion was used with a time window of 45 s. The single charged peaks and peaks with charges exceeding 6 were masked and excluded from further analyses.

### Proteome discoverer database search

The vendor’s raw MS files were processed using Proteome Discoverer (PD) software (Version 2.4.0.305) and the built-in Sequest HT search engine according to the description with some modifications [33]. MS spectra lists were searched against their species-level UniProt FASTA databases Uniprot (Bacillus subtilis_1423-2021–09.fasta), with Carbamidomethyl [C], TMT 6 plex (K) and TMT 6 plex (N-term) as a fixed modification and oxidation (M) and acetyl (Protein N-term) as variable modifications. Trypsin was used as proteases. A maximum of 2 missed cleavage(s) was allowed. The false discovery rate was set to 0.01 for PSM and peptide levels. Peptide identification was performed with an initial precursor mass deviation of up to 10 ppm and a fragment mass deviation of 0.02 Da. Unique peptide and razor peptide were used for protein quantification and total peptide amount for normalization. All the other parameters were reserved as default.

### Metabolites extraction

The metabolites extraction according to the methods described with minor modifications [33, 34]. Briefly, a 500 μL PBS was added to the sample in a 2 mL EP tube. After centrifugation at 4℃ for 10 min at 5,000 rpm, all supernatants were carefully removed into a 2 mL EP tube and placed in a refrigerator at -80℃. The precipitate was then weighed in a 2 mL centrifuge tube, and 200 μL pure water was added, followed by vortex mixing for 30 s. Next, the steel ball was added and processed with a 40 Hz grinding instrument for 4 min, followed by ultrasonic treatment with an ice water bath for 5 min (repeat three times). Next, 20 μL homogenate was taken for BCA, 165 μL methanol and 115 μL precooled chloroform was added, and vortexed for 30 s. Ultrasound was used for 10 min (ice bath). Afterwards, the 250 μL supernatant was transferred to a new tube after centrifugation at 12,000 rpm for 15 min (4℃). To prepare the quality control sample, 80 μL supernatant of each sample was combined together. Subsequently, the supernatant was dried in a vacuum concentrator, and then 40 μL methoxyamination hydrochloride (20 mg/mL in pyridine) was added and cultured at 80℃ for 30 min. Next, 50 μL of the N,O-Bis(trimethylsilyl)trifluoroacetamide (BSTFA) regent with 1% trimethylchlorosilane (TMCS, v/v) was added and incubated for 1.5 h at 70℃. Samples were gradually cooled to room temperature. All samples were then analyzed by gas chromatography coupled with a time-of-flight mass spectrometer.

### *GC* × *GC-TOF–MS analysis*

Agilent 7890 gas chromatograph system coupled with a Pegasus 4D time of flight mass spectrometer was used for GC × GC-TOF–MS analysis according to the methods described by previous report with minor modifications [33–35]. A 1 μL aliquot of the analyte was injected in splitless mode. The carrier gas used was helium and the front inlet purge flow was 3 mL min^−1^and the gas flow rate through the column was 1 mL min^−1^. The column temperature was programmed as follow: the initial temperature at 90 °C (held for 1 min), increased to 180 °C at 10 °C min^−1^ (held for 1 min), increased to 240 °C at 5 °C min^−1^ (held on 1 min), increased to 300 °C at 30 °C min^−1^ (held on 12 min). The injection, transfer line and ion source temperatures were set at 280, 250, and 220 °C respectively. The ionisation mode was operated in electron impact (electron energy -70 eV). The mass spectrometry data were acquired in the full-scan mode at a rate of 100 spectra per second using a mass scan range of 33–550 m/z after a solvent delay of 5.85 min.

### Data preprocessing and annotation

Chroma TOF software of LECO Corporation and Fiehn database were used for raw peaks exacting, the data baselines filtering and calibration of the baseline, peak alignment, deconvolution analysis, peak identification, and integration of the peak area [36]. Next, the DEGs, differential expression proteins, and metabolites were integrated and analyzed by multi-omics using the following conditions: *P*-value < 0.05 and |log_2_fold change (FC) |≥ 1.

## Results

### Effect of culture temperature on flavor production

In the present study, BJ3-2 was selected as the target strain, whose growth and physiological characterization was investigated by culturing in LB medium at different temperatures. The results showed that the strain cultured at 53℃ had a darker color compared to that cultured at 37℃, while only a slight color difference was observed at 45℃ (Fig. S1A). Moreover, the strain cultured at 45℃ and 53℃ had a lower OD value than that cultured at 37℃ (Fig. S1B). Under the biologic photomicroscope, the bacteria morphology of BJ3-2 strain cultured at 37℃, 45℃, and 53℃ were observed to be rod-shaped, and the gram staining showed that the strain was gram-positive bacterium (Fig. S1C-E). Since the significant variation of fermentation broth in color was observed at the different temperatures, especially at 53℃, the produced flavor was further evaluated. As shown in Table 1, fermentation without strains inoculated in the LB medium exhibited only sweet flavor, indicating that the medium would not produce the target flavor at high temperatures. Moreover, the strain cultured at 37℃ had increased the soybean flavor (a special flavor of ammonia-containing smelly distinct from natto) compared with the strain cultured at 45℃ and 53℃. On the contrary, the strain cultured at 45℃ and 53℃ had a higher soy sauce-like flavor than that cultured at 37℃. These results suggested that the BJ3-2 has various metabolic properties in different temperatures. The observation was in agreement with the previous studies reporting that the temperature was crucial for soy sauce-like flavor formation in Heat-Resistant Strain *Bacillus licheniformis* CGMCC3962 [26]. Consequently, the growth and physiological difference of *Bacillus subtilis* BJ3-2 indicated that the metabolic regulation was not constant at the various temperatures.

**Table 1:** Effect of culture temperature on flavor production

Strain	Sensory evaluation		
	37℃	45℃	53℃
None	Sweet	Sweet	Sweet
BJ3-2	Soybean flavor	Soybean /soy sauce flavor	Soy sauce flavor

### Transcriptome analysis of the AT37, BT45, and CT53 samples

**Fig. 1:**
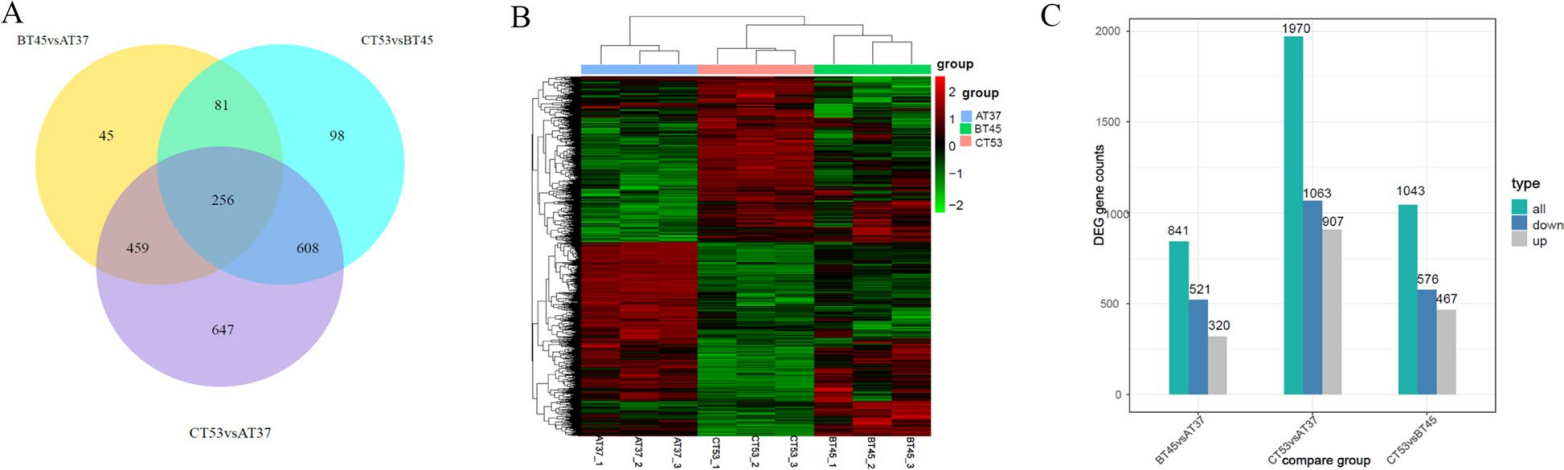
Transcriptome analysis of the BJ3-2 cultured at 37 °C, 45 °C and 53 °C. **A** Venn diagram indicating the differential gene expression in the AT37, BT45 and CT53; **B** Heat map analysis of DEGs of the AT37, BT45 and CT53; **C** The DEGs of the AT37, BT45 and CT53

To better understand the role of genes involved in physiological metabolism at the molecular level, a comparative transcriptome analysis was performed using the samples of AT37, BT45, and CT53. In total, 71 169 628 raw reads were generated from 9 samples (AT37_1/2/3, BT37_1/2/3, CT37_1/2/3), including 23 885 294 raw reads from AT37 samples, 24 158 336 raw reads from AT45 samples, and 23 125 998 raw reads from AT53 samples. The raw sequences were filtered, and 23 738 006, 24 012 568, and 22 974 490 clean reads were generated for AT37, BT45, and CT53, respectively (Table S1). Considering the sequencing data, the detected percentages were within a reasonable range (Table S1). Furthermore, the gene expression was detected in the AT37, BT45, and CT53 samples with FPKM > 0, and the gene expression levels in different samples were uniformly distributed in density diagrams (Fig. S2). Regarding the expression of these genes, the three biological replicates for RNA-Seq were applied, and the results showed correlation coefficients > 0.898 (Fig. S3A). Analogously, the principal component analysis (PCA) was obtained from the data indicating a distinct separation among the samples of AT37, BT45, and CT53 for the same line (Fig. S3B). These results suggested that the RNA data for the three biological replicates displayed good reproducibility and consistency between replicates. Besides, 256 DEGs were identified in all of the groups, 45 DEGs occurred only in the BT45 of BT45vsAT37, 647 DEGs occurred only in CT53 of CT53vsAT37, and 98 DEGs occurred only in CT53 of CT53vsBT45 (Fig. 1A). The DEGs were subsequently analyzed, and a clustering heat map was drawn to show the significant differences (Fig. 1B). Therefore, the transcript levels of each gene in the data of RNA-seq were analyzed using an adjusted *P*-value < 0.05 and |log2FC|≥ 1 as the significance. As shown, 841 DEGs were identified in BT45 vs. AT37; among them, there were 521 down-regulated DEGs and 320 up-regulated DEGs. In comparison with the DEGs of AT37 and CT53, 1970 DEGs were identified; among them, there were 1063 down-regulated DEGs and 907 up-regulated DEGs. Similarly, 1043 DEGs were identified in CT53 vs. BT45; among them, there were 576 down-regulated DEGs and 467 up-regulated DEGs (Fig. 1C, Fig. S4A-C, Table S2). For comparison, the DEGs of AT37, BT45, and CT53 samples were classified into four clusters (1–4) according to their expression patterns, and similar expression trends for the DEGs were observed in each cluster (Fig. S4D).

### GO and KEGG enrichment of the DEGs

**Fig. 2:**
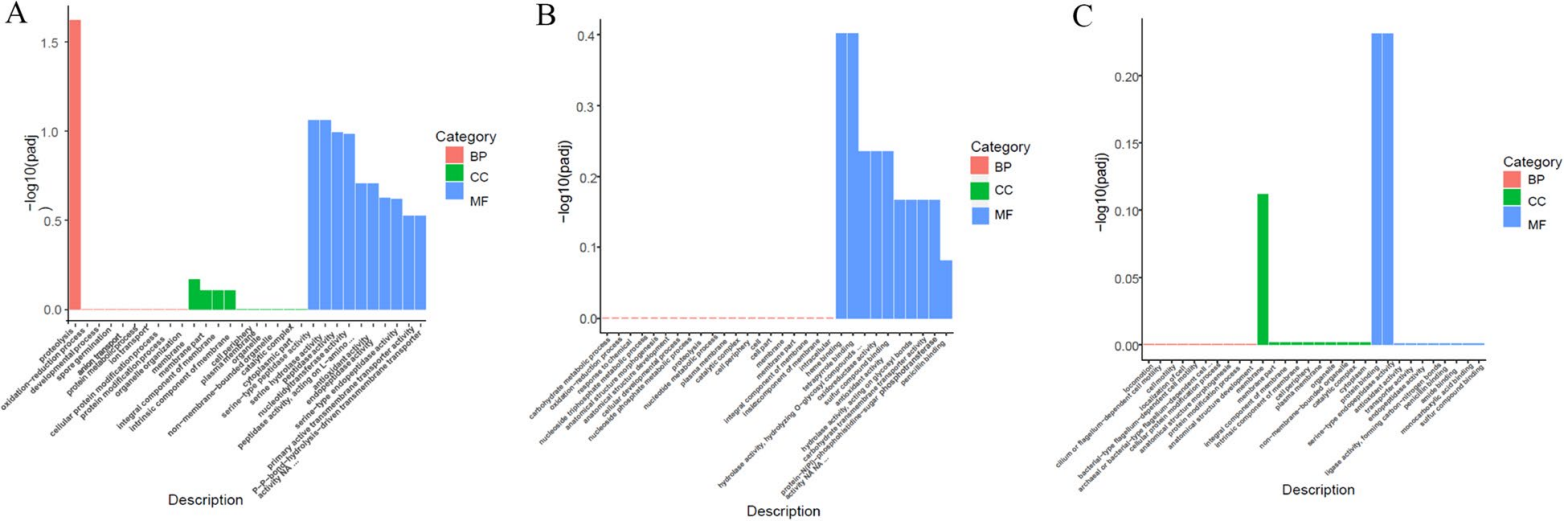
GO enrichment analysis of DEGs. **A** GO enrichment analysis of DEGs in AT37 and BT45; **B** GO enrichment analysis of DEGs in AT37 and CT53; **C:** GO enrichment analysis of DEGs in BT45 and CT53

**Fig. 3:**
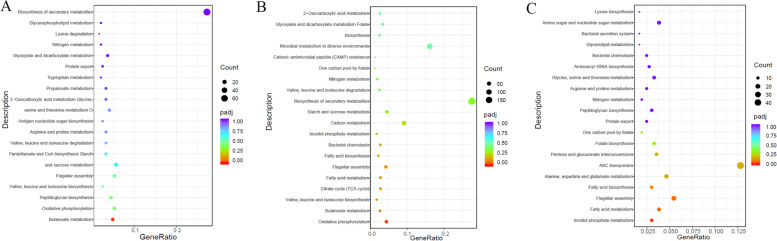
KEGG pathway analysis of DEGs. **A** KEGG pathway analysis of DEGs in AT37 and BT45; **B** KEGG pathway analysis of DEGs in AT37 and CT53; **C** KEGG pathway analysis of DEGs in BT45 and CT53

The functions of the DEGs were identified using GO classification based on their roles in biological process (BP), molecular function (MF), and cellular component (CC). All the DEGs in BT45 vs. AT37 were dominantly categorized into the MF categories, and most of the DEGs were connected to serine-type peptidase activity and serine hydrolase activity (Fig. 2A, Table S3). When comparing CT53 with AT37, most DEGs were enriched to heme binding and tetrapyrrole binding (Fig. 2B, Table S4). However, the DEGs in CT53 vs. BT45 were categorized into the two main categories (CC and MF), and most of the DEGs were enriched to the membrane and protein binding and serine-type endopeptidase activity (Fig. 2C, Table S5). Meanwhile, KEGG analysis showed that most of the DEGs in BT45 vs. AT37 and CT53 vs. AT37 were enriched in pathways related to the biosynthesis of secondary metabolites (Fig. 3A, B, Table S6, 7). However, the pathways of DEGs in CT53 vs. BT45 were closely enriched in the ABC transporters (Fig. 3C, Table S8), which is consistent with the amino acid transport and metabolism in *Deinococcus radiodurans* [37].

### Proteomic analysis of the AP37, BP45, and CP53 samples

**Fig. 4:**
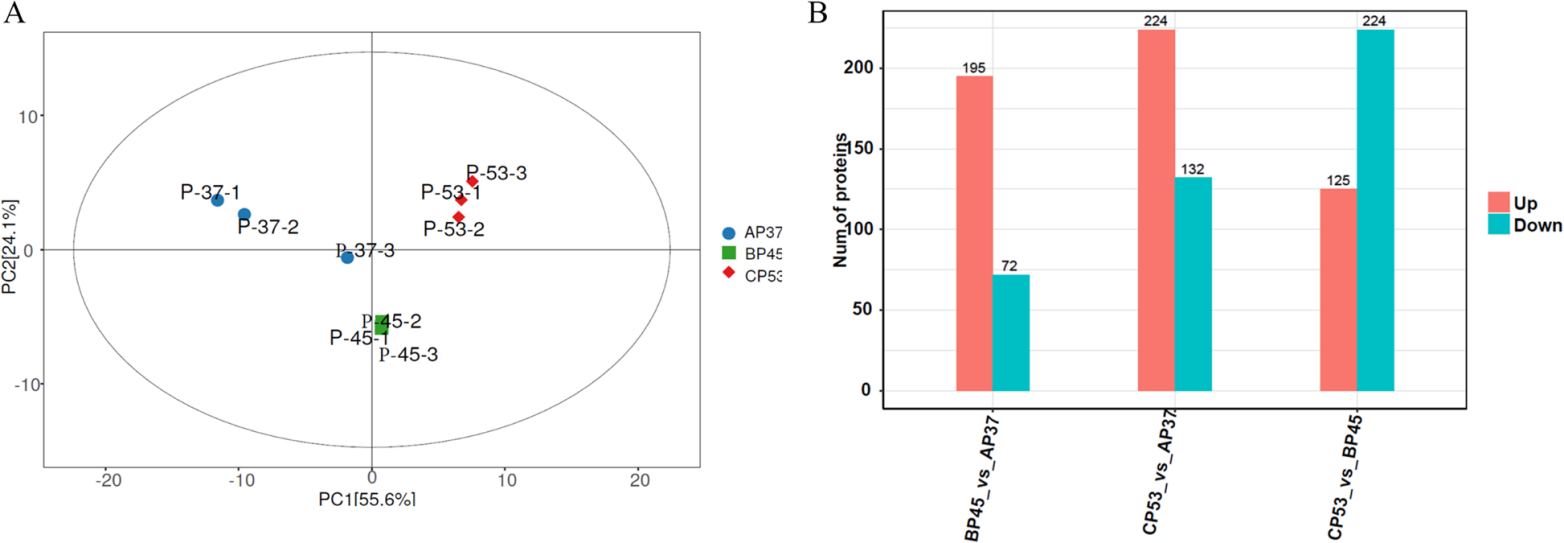
Proteomic analysis of the AP37, BP45 and CP53. **A** Correlation analysis of the AP37, BP45 and CP53; **B** The DEPs of the AP37, BP45 and CP53

To identify the changes involved in physiological metabolism among the samples of AP37, BP45 and CP53 in proteomic levels, the proteins samples were extracted and subjected to electrophoresis in an SDS–polyacrylamide gel. The results showed a good overall quality of the protein structure (Fig. S5). Next, a comparative proteomic analysis was performed using the samples of AP37, BP45, and CP53. The principal component analysis (PCA) showed a distinct separation among the samples of AP37, BP45, and CP53 for the same line (Fig. 4A), which suggested that the RAW data for the three biological replicates displayed good reproducibility and consistency between replicates. Therefore, the protein levels of each RAW in the data of proteomic analysis were defined as the significance (*P*-value < 0.05 and |log2FC|≥ 1). As shown, 72 down-regulated and 195 up-regulated proteins were identified in BP45 vs. AP37. Similarly, 132 down-regulated and 224 up-regulated proteins were found when comparing AP37 with CP53. Likewise, 224 down-regulated and 125 up-regulated proteins were identified in CP53 vs. BP45 (Fig. 4B, Table S9). The differentially expressed proteins (DEPs) were subsequently analyzed, and the clustering heat map and volcano plot were drawn to show the significant differences (Fig. S6, 7).

### Enrichment of the DEPs

**Fig. 5:**
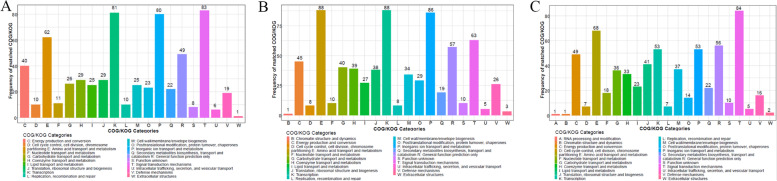
COG analysis of DPGs. **A** COG analysis of DPGs in AP37 and BP45; **B** COG analysis of DPGs in AP37 and CP53; **C:** COG analysis of DPGs in BP45 and CP53

**Fig. 6:**
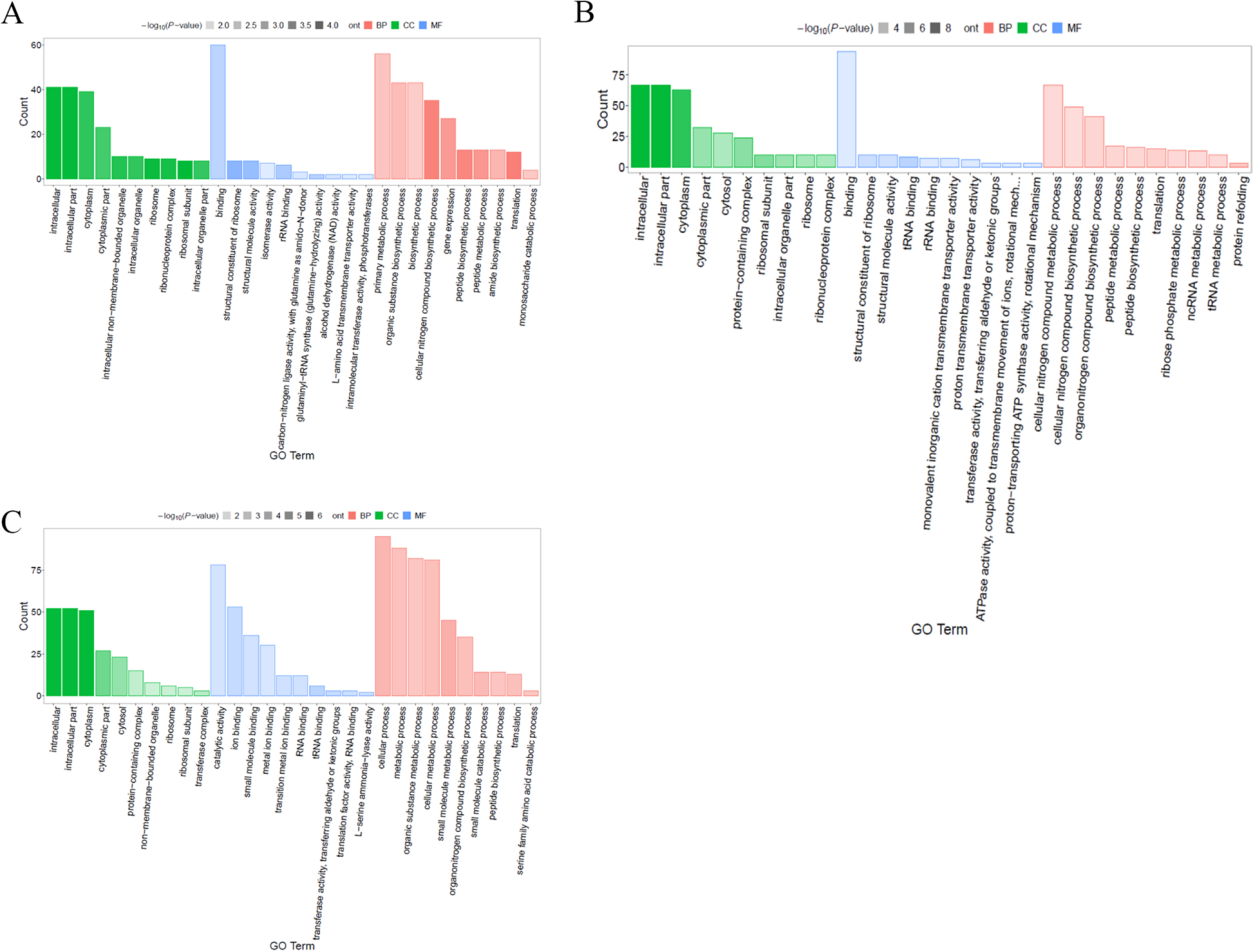
GO enrichment analysis of DPGs. **A** GO enrichment analysis of DPGs in AP37 and BP45; **B** GO enrichment analysis of DPGs in AP37 and CP53; **C** GO enrichment analysis of DPGs in BP45 and CP53

**Fig. 7:**
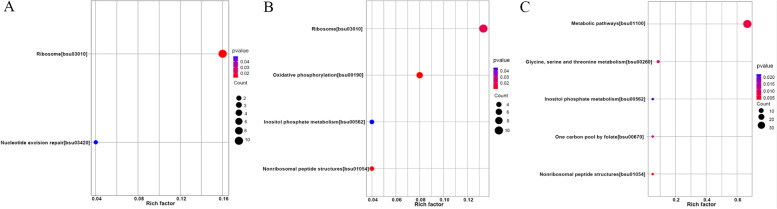
KEGG pathway analysis of DPGs. **A** KEGG pathway analysis of DPGs in AP37 and BP45; **B** KEGG pathway analysis of DPGs in AP37 and CP53; **C** KEGG pathway analysis of DPGs in BP45 and CP53

The frequency of the DEPs was matched using COG (Cluster of Orthologous Groups of proteins) analysis (Fig. 5). Interestingly, we found that most DEPs could be dominantly matched to five COG functional categories (amino acid transport and metabolism, transcription, inorganic ion transport and metabolism, general function prediction, and signal transduction mechanisms). Moreover, all the DEPs in BT45 vs. AT37 were mainly classified into MF categories. Additionally, the functions of the DEPs were identified using GO analysis. As shown in Fig. 6 and Table S10, 11, 12, all the DEPs in BP45 vs. AP37, CP53 vs. AP37, and CP53 vs. BP45 were categorized into three main categories (BP, CC, and MF)(Fig. 6A-C). For CC, most DEPs in BP45 vs. AP37, CP53 vs. AP37, and CP53 vs. BP45 were enriched in intracellular, intracellular part and cytoplasm. For BP, the DEPs in BP45 vs. AP37, CP53 vs. AP37, and CP53 vs. BP45 were primarily enriched in the primary metabolic process, organic substance biosynthetic process, and biosynthetic process. For MF, most of the DEPs in BP45vsAP37 were connected to the binding **(**Fig. 6A). However, most of the DEPs in BP45 vs. AP37 and CP53 vs. BP45 were connected to the catalytic activity (Fig. 6B, C). Meanwhile, KEGG analysis showed that most of the DEPs in BP45 vs. AP37 were enriched in pathways related to ribosome and nucleotide excision repair (Fig. 7A). Similarly, most of the DEPs in CP53 vs. AP37 were enriched in pathways related to oxidative phosphorylation, nonribosomal peptide structures, ribosome, and inositol phosphate metabolism (Fig. 7B). Notably, KEGG enriched analysis revealed that pathways closely related to the glycine, serine and threonine metabolism were found in CP53 vs. BP45 (Fig. 7C), which is consistent with the high accumulation of NH_4_^+^ and pyruvic acid in *Bacillus subtilis*.

### Overview of the metabolic profiling

**Fig. 8:**
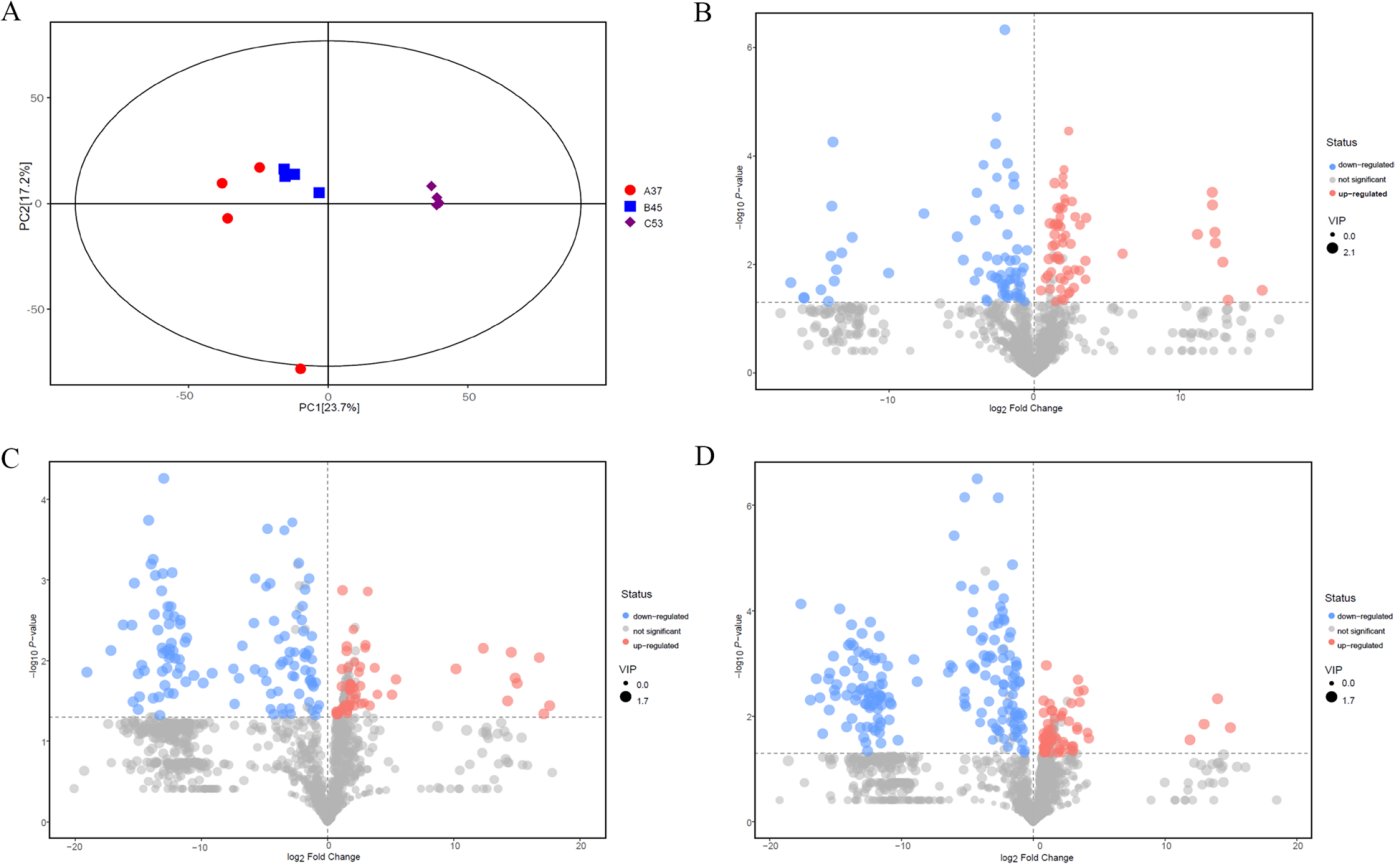
Metabolomics analysis of the metabolites. **A** Score scatter plot of PCA model for A37, B45 and C53; **B** Volcano plot for group A37 vs B45; **C** Volcano plot for group A37 vs C53; **D** Volcano plot for group B45 vs C53

**Fig. 9:**
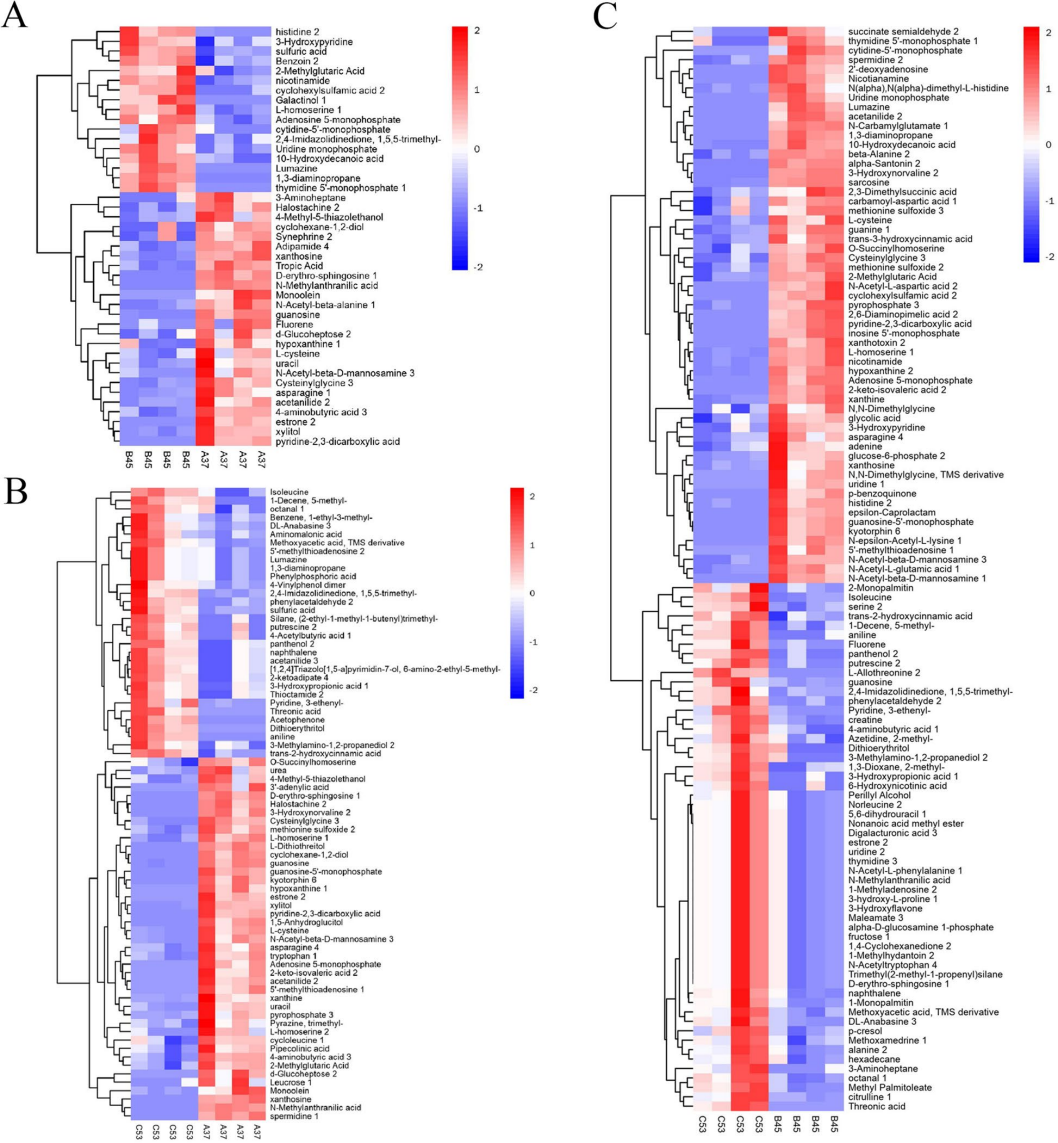
Heat map analysis of the metabolites. **A** Heat map analysis of the metabolites for group A37 vs B45; **B** Heat map analysis of the metabolites for group A37 vs C53; **C** Heat map analysis of the metabolites for group B45 vs C53

**Fig. 10:**
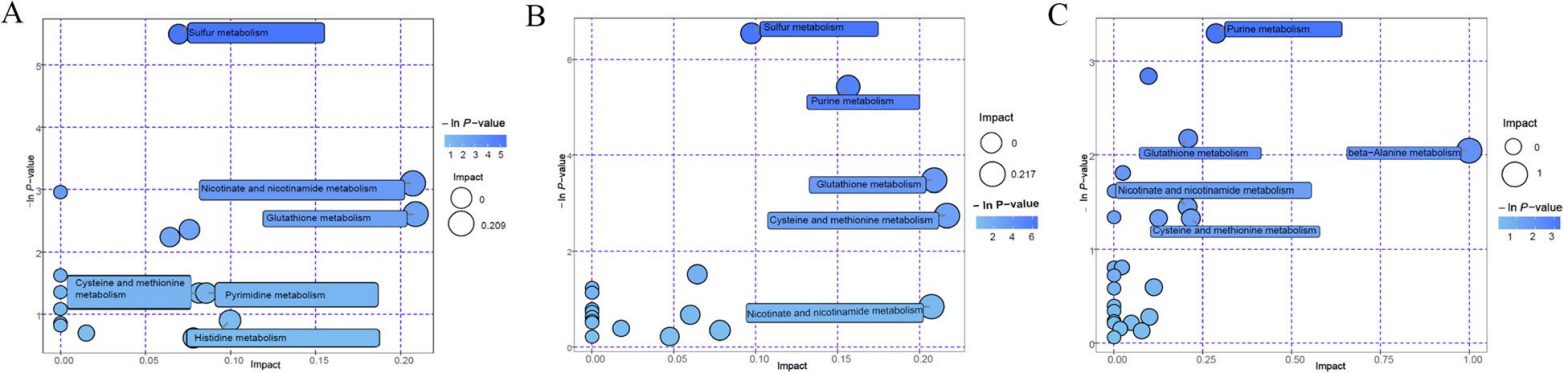
Pathway analysis.** A** Pathway analysis for group A37 vs B45; **B** Pathway analysis for group A37 vs C53; **C** Pathway analysis for group B45 vs C53

To further compare the metabolite compositions in A37, B45, and C53, the samples of these strains were subjected to GC × GC-TOF–MS analysis. The score scatter plot for the PCA model indicated that the samples were clearly isolated within the PC1 × PC2 score plots (Fig. 8A). The volcano plot of the metabolites content showed significant differences in every four samples (Fig. 8B-D). Among them, 64 different metabolites were markedly up-regulated, and 68 were down-regulated in BM45 compared to those in A37. Additionally, 68 different metabolites were markedly up-regulated, and 114 were down-regulated in C53 vs. A37. Moreover, 130 different metabolites were markedly up-regulated and 169 were down-regulated in C53 vs. B45 (Table S13). Hierarchical clustering analysis of those different metabolites was performed, and a heat map was drawn showing the different patterns in the A37, B45, and C53 (Fig. 9A-C). Moreover, a significant metabolic difference was detected among the A37, B45, and C53 samples. Based on the differences in metabolites, a metabolic network indicating the compound involved pathways in B45 vs. A37 and C53 vs. A37 revealed that they were enriched in metabolism pathways, including sulfur metabolism, glutathione metabolism, nicotinate, and nicotinamide metabolism, cysteine and methionine metabolism, and pyrimidine metabolism (Fig. 10A,B, Fig. S8). In addition to the above metabolisms, beta-alanine metabolism was also enriched in C53 vs. B45 (Fig. 10C).

## Discussion

*B. subtilis* is an important microorganism widely used in the food industry [5]. Some foods fermented by *B. subtilis* have functional ingredients that improve nutrition and health [7, 8, 23]. Currently, there are numerous studies on *B. subtilis,* which provide an excellent source of data related to valuable favor/nutrients compounds [9, 10]. Nevertheless, to date, no study reported comprehensive identification of soy sauce-like flavor and soybean flavor compound accumulation in *B. subtilis*.

It is generally considered that the growth and metabolism at different temperatures have variability patterns in microorganisms [23, 38]. In the present study, we investigated the growth and physiological characterization of *B. subtilis* BJ3-2, finding that BJ3-2 cultured at different temperatures had various phenotypes on color (Fig. S1A, B). BJ3-2 cultured at 53℃ had a darker color compared with that cultured at 37℃, but only a slight difference in colors when cultured at 45℃ (Fig. S1A). These results indicated that temperature affects the color of fermentation broth, thus suggesting a potential relationship with the formation of soy sauce-like flavor and soybean flavor. In addition, the strain cultured at 45℃ and 53℃ had a lower OD value than that cultured at 37℃ (Fig. S1B). Previous studies demonstrated that the temperature affected soy sauce-like flavor formation in heat-resistant strain *Bacillus licheniformis* [26]. We found that the strain cultured at 37℃ had increased soybean flavor compared with strains cultured at 45℃ and 53℃. Interestingly, the strain cultured at 45℃ and 53℃ had a higher soy sauce-like flavor than that cultured at 37℃ (Table 1). Therefore, these results suggested that BJ3-2 has various metabolic properties at different temperatures, which could produce soy sauce-like flavor at a high temperature and soybean flavor at a lower temperature. These results are in agreement with the brewing process of Maotai flavor liquor, where the formation of Maotai flavor was kept around high fermentation temperature (55 − 62 °C).

To investigate the molecular mechanisms of the formation of soy sauce-like and soybean flavor in the BJ3-2, a transcriptome analysis was performed, and the DEGs between groups were identified (Fig. 1A-C, Fig. S4A-C). Furthermore, KEGG analysis showed that most of the DEGs in BT45 vs. AT37 and CT53 vs. AT37 were enriched in pathways related to the biosynthesis of secondary metabolites (Fig. 3A, B). In addition, the results showed that the transcripts of metabolism-related genes (*yisZ, yitA, mmgD, asnO, gapA, proB, proA, mccB, lpdV, thrB, thrD,* and *racX*) changed in the BT45 and CT53 compared to AT37 (Table S14). However, the pathways of DEGs in CT53 vs. BT45 were closely enriched in the ABC transporters (Fig. 3C), which is consistent with the amino acid transport and metabolism in *Deinococcus radiodurans* [37]. Consistently, similar results showed that the expressions of ABC transporters related genes (*rmgP, pstA, opuBB, msmE, opuBA, opuCD, opuCC, msmF, opuCB, msmX, opuBD, opuCA, bioY, yxeO, yxeM,* and *msmG*) were significantly changed in the CT53 compared to BT45 (Table S14). Our results suggested that the changes in expression levels of amino acid metabolism-related genes would induce flavor substances accumulation in the BJ3-2 strain.

*B. subtilis* is one of the most dominant microorganisms found in fermented foods [5]. Numerous studies have addressed *B. subtilis*, representing an excellent source of data related to valuable nourishing, functional food ingredients and flavor compounds [5, 7–9, 11]. Nevertheless, no study on comprehensive identification of the knowledge of soy sauce-like flavor and soybean flavor on key compounds in *Bacillus subtilis*. Herein, BJ3-2 strain was first subjected to proteomic analysis. As shown, 267 DEPs (72 down-regulated and 195 up-regulated), 356 DEPs (132 down-regulated and 224 up-regulated), and DEPs (224 down-regulated and 125 up-regulated) were identified in BP45 vs. AP37, CP53 vs. AP37, and CP53 vs. BP45, respectively (Fig. 4B). Interestingly, we found that most DEPs could be dominantly matched to amino acid transport and metabolism (Figs. 5, 6 and 7), which are consistent with the high accumulation of NH_4_^+^ and pyruvic acid in *B. subtilis*. It has been shown that 64 metabolites were markedly up-regulated, and 68 were down-regulated in B45 compared to those in A37 (Fig. 8B). Additionally, 68 metabolites were markedly up-regulated, and 114 were down-regulated in C53 compared to those in A37 (Fig. 8C). Moreover, 130 metabolites were markedly up-regulated and 169 were down-regulated in C53 compared to those in B45 (Fig. 8D). These results indicated great metabolic differences in the two samples. Furthermore, KEGG analysis has shown that the most differential metabolites were enriched in various metabolism pathways, including sulfur metabolism, cysteine and methionine metabolism, and pyrimidine metabolism (Fig. 10A, B, Fig. S8). Correspondingly, L-homoserine, L-homoserine, L-cysteine, sulfuric acid, O-Succinylhomoserine, and beta-alanine had significant changes in B45 and C53 compared with that in A37 (Table S14). Notably, the sulfur-containing aroma compounds are key contributors to the flavor of a diverse range of foods and beverages [27], which was remarkably changed in B45 and C53. Moreover, the compounds that participated in the cysteine and methionine metabolism and pyrimidine metabolism pathway also revealed obvious differences in the B45 and C53 compared to A37. Consistently, a stronger soy sauce-like flavor was also observed in the strain cultured at 45℃ and 53℃, while a stronger soybean flavor was found in the strain cultured at 37℃ (Table 1). These results suggested potential changes in sulfur metabolism, cysteine and methionine metabolism, and pyrimidine metabolism in the strains cultured at 45℃ and 53℃, even affecting the higher soy sauce-like flavor phenotype.

## Conclusions

In summary, we combined transcriptomics, proteomics, and metabolomics analyses to explore the mechanisms underlying the flavor accumulation in *B. subtilis* at different temperatures. Transcriptome analysis revealed more than 841 DEGs, 1970 DEGs, and 1043 DEGs that participated in soy sauce-like and soybean flavor accumulation in BT45 vs. AT37, CT53 vs. AT37, and CT53 vs. BT45, respectively. Moreover, the significantly changed metabolites involved in sulfur metabolism, cysteine and methionine metabolism, and pyrimidine metabolism in the strains cultured at 45℃ and 53℃ may cause soy sauce-like and soybean flavor accumulation in *B. subtilis* BJ3-2. These results further our understanding of the mechanism of soy sauce-like flavor and soybean flavor in *B. subtilis.* They also provide a series of candidate genes and metabolic engineering to enhance the production of flavor compounds.

## Supplementary Information

**Additional file 1:** **Fig. S1**. Growth and physical characteristics. **A:** The color of fermentation broth; **B:** The OD value of fermentation broth; **C:** Gram staining of BJ3-2 cultured at 37°C; **D:** Gram staining of BJ3-2 cultured at 45°C; **E:** Gram staining of BJ3-2 cultured at 53°C*. ***Fig. S2***. *Expression density distribution. **Fig. S3**. Data quality control of RNA-seq. **A:**Pearson correlation between smaples; **B:** Principal component analysis. **Fig. S4**. Analysis of DEGs. **A:** Volcano plot for group AT37 vs BT45; **B:** Volcano plot for group AT37 vs CT53; **C:**Volcano plot for group BT45 vs CT53; **D: **Cluster analysis of DEGs using H-cluster method. **Fig. S5**. Electrophoretograms of protein expressions. **Fig. S6**. Heat map analysis of DPGs in the AP37 vs BP45 **A**, AP37 vs CP53 **B** and BP45vs CP53 **C**. **Fig. S6**. Heat map analysis of DPGs in the AP37 vs BP45 **A**, AP37 vs CP53 **B** and BP45vs CP53 **C**. **Fig. S7**. Volcano plot analysis of DEPs in the AP37 vs BP45 **A**, AP37 vs CP53 **B** and BP45vs CP53**C**. **Fig. S8**.KEGG enrichment analysisfor group A37 vs B45 **A**, A37 vs C53 **B** and B45 vs C53 **C**.

**Additional file 2:** **Table S1.** The data set summary of transcriptome sequence.

**Additional file 3:** **Table S2.** The summary of DEGs.

**Additional file 4: Table S3.** GO enrich for BT45vsAT37. 

**Additional file 5:** **Table S4.** GO enrich for CT53vsAT37.

**Additional file 6: Table S5.** GO enrich for CT53vsBT45

**Additional file 7:** **Table S6.** KEGG enrich for BT45vsAT37.

**Additional file 8:** **Table S7.** KEGG enrich for CT53vsAT37.

**Additional file 9:** **Table S8.** KEGG enrich for CT53vsBT45.

**Additional file 10:** **Table S9.** List of DEPs. 

**Additional file 11: Table S10. **GO annotation enrichment analysis in BP45_vs_AP37. 

**Additional file 12:** **Table S11.** GO Annotation Enrichment Analysis in CP53_vs_AP37

**Additional file 13:** **Table S12.** GO Annotation Enrichment Analysis in CP53_vs_BP45.

**Additional file 14: Table S13. **Differentially expressed metabolites.

**Additional file 15:** **Table S14.** Information of correlation analysis by multiple omics.

## Data Availability

All generated or analysed data (including figures and tables) during this study were not submitted elsewhere and are included in this published article. Supplementary figures and tables to this article can be found in the Supplementary Materials. All datasets are available from the corresponding author on reasonable request.

## References

[1] Liu Y, Cheng H, Liu H, Ma R, Ma J, Fang H (2019). Fermentation by Multiple Bacterial Strains Improves the Production of Bioactive Compounds and Antioxidant Activity of Goji Juice. Molecules.

[2] Talib N, Mohamad NE, Yeap SK, Hussin Y, Aziz MNM, Masarudin MJ, Sharifuddin SA, Hui YW, Ho CL, Alitheen NB: Isolation and Characterization of Lactobacillus spp. from Kefir Samples in Malaysia. *Molecules* 2019, 24(14):2606. 10.3390/molecules24142606 PMC6680525 31319614

[3] Song JL, Choi JH, Seo JH, Lim YI, Park KY (2014). Anti-colitic effects of kanjangs (fermented soy sauce and sesame sauce) in dextran sulfate sodium-induced colitis in mice. J Med Food.

[4] Zhou K, Siroli L, Patrignani F, Sun Y, Lanciotti R, Xu Z (2019). Formation of Ethyl Carbamate during the Production Process of Cantonese Soy Sauce. Molecules.

[5] Zhi Y, Wu Q, Du H, Xu Y: Biocontrol of geosmin-producing Streptomyces spp. by two Bacillus strains from Chinese liquor. International journal of food microbiology 2016, 231:1–9. 10.1016/j.ijfoodmicro.2016.04.021 27161758

[6] Yan Z (2013). Zheng Xe, Chen Ju, Han Jh, Han Bo: Effect of different Bacillus strains on the profile of organic acids in a liquid culture of Daqu. Asymmetric Michael Addition of an Alanine Derivative.

[7] Wen  A, Qin  L , Zeng  H , Zhu  Y (2020). Comprehensive evaluation of physicochemical properties and antioxidant activity of B. subtilis-fermented polished adlay subjected to different drying methods. Food science & nutrition.

[8] Wen A, Xie C, Mazhar M, Zhu Y, Zhu Y: Comparative evaluation of drying methods on kinetics, biocompounds and antioxidant activity of Bacillus subtilis-fermented dehulled adlay. Drying Technology 2019:1–11.

[9] Liang H, Li W, Luo Q, Liu C, Wu Z, Zhang W (2015). Analysis of the bacterial community in aged and aging pit mud of Chinese Luzhou-flavour liquor by combined PCR-DGGE and quantitative PCR assay. J Sci Food Agric.

[10] Lin Q, Xiao Z, Qiuxiang FU, Fang X, Longjiu MO, Pan Q, Coltd DD: Isolation of Aroma-producing Bacillus licheniformis and Analysis of Its Fermentation Metabolites. Liquor-Making Science & Technology 2013.

[11] Ramos CL, de Almeida EG, Freire AL, Freitas Schwan R (2011). Diversity of bacteria and yeast in the naturally fermented cotton seed and rice beverage produced by Brazilian Amerindians. Food Microbiol.

[12] Zhu  BF, Xu  Y, Fan  WL (2010). High-yield fermentative preparation of tetramethylpyrazine by Bacillus sp. using an endogenous precursor approach. Journal of industrial microbiology & biotechnology.

[13] Buttery RG, Orts WJ, Takeoka GR, Nam Y (1999). Volatile flavor components of rice cakes. J Agric Food Chem.

[14] Xiao ZJ, Xie NZ, Liu PH, Hua DL, Xu P (2006). Tetramethylpyrazine production from glucose by a newly isolated Bacillus mutant. Appl Microbiol Biotechnol.

[15] Xu W, Xu Q, Chen J, Lu Z, Xia R, Li G, Xu Z, Ma Y (2011). Ligustrazine formation in Zhenjiang aromatic vinegar: changes during fermentation and storing process. J Sci Food Agric.

[16] Kosuge T, Adachi T, Kamiya H (1962). Isolation of tetramethylpyrazine from culture of Bacillus natto, and biosynthetic pathways of tetramethylpyrazine. Nature.

[17] Kitagawa M, Shiraishi T, Yamamoto S, Kutomi R, Ohkoshi Y, Sato T, Wakui H, Itoh H, Miyamoto A, Yokota SI (2017). Novel antimicrobial activities of a peptide derived from a Japanese soybean fermented food, Natto, against Streptococcus pneumoniae and Bacillus subtilis group strains. AMB Express.

[18] Fan W, Xu Y, Zhang Y (2007). Characterization of pyrazines in some Chinese liquors and their approximate concentrations. J Agric Food Chem.

[19] Larroche C, Besson I, Gros JB (1999). High pyrazine production by Bacillus subtilis in solid substrate fermentation on ground soybeans. Process Biochem.

[20] Xiao Z, Lu JR (2014). Generation of acetoin and its derivatives in foods. J Agric Food Chem.

[21] Xiao Z, Lu JR (2014). Strategies for enhancing fermentative production of acetoin: a review. Biotechnol Adv.

[22] Meng W, Xiao D, Wang R (2016). Enhanced production of tetramethylpyrazine in Bacillus licheniformis BL1 by bdhA disruption and 2,3-butanediol supplementation. World J Microbiol Biotechnol.

[23] Xu Y, Jiang Y, Li X, Sun B, Teng C, Yang R, Xiong K, Fan G, Wang W (2018). Systematic Characterization of the Metabolism of Acetoin and Its Derivative Ligustrazine in Bacillus subtilis under Micro-Oxygen Conditions. J Agric Food Chem.

[24] Cui DY, Wei YN, Lin LC, Chen SJ, Feng PP, Xiao DG, Lin X, Zhang CY (2020). Increasing Yield of 2,3,5,6-Tetramethylpyrazine in Baijiu Through Saccharomyces cerevisiae Metabolic Engineering. Front Microbiol.

[25] Zhu S, Lu X, Ji K, Guo K, Li Y, Wu C, Xu G (2007). Characterization of flavor compounds in Chinese liquor Moutai by comprehensive two-dimensional gas chromatography/time-of-flight mass spectrometry. Anal Chim Acta.

[26] Wu Q, Xu Y (2012). Transcriptome profiling of heat-resistant strain Bacillus licheniformis CGMCC3962 producing Maotai flavor. J Agric Food Chem.

[27] Holt  S , Cordente  AG, Williams  SJ, Capone  DL, Jitjaroen  W , Menz  IR, Curtin  C,  Anderson  PA (2011). Engineering Saccharomyces cerevisiae to release 3-Mercaptohexan-1-ol during fermentation through overexpression of an S. cerevisiae Gene, STR3, for improvement of wine aroma. Applied and environmental microbiology.

[28] Fan W, Shen H, Xu Y (2011). Quantification of volatile compounds in Chinese soy sauce aroma type liquor by stir bar sorptive extraction and gas chromatography-mass spectrometry. J Sci Food Agric.

[29] 贾东旭, 吴拥军, 李耀中, 许文钊: 细菌型豆豉发酵芽孢杆菌的筛选与鉴定. *食品科学* 2009(5):5.

[30] Wen A, Xie C, Mazhar M, Wang C, Zeng H, Qin L, Zhu Y (2020). Tetramethylpyrazine from adlay (Coix lacryma-jobi) biotransformation by Bacillus subtilis and its quality characteristics. J Food Sci Technol.

[31] Kanehisa M, Furumichi M, Sato Y, Ishiguro-Watanabe M, Tanabe M (2021). KEGG: integrating viruses and cellular organisms. Nucleic Acids Res.

[32] Liu D, Zhang L, Wang Y, Li Z, Wang Z, Han J (2020). Effect of high hydrostatic pressure on solubility and conformation changes of soybean protein isolate glycated with flaxseed gum. Food Chem.

[33] Ma H, Lai BJ, Jin YF, Tian C, Liu JY, Wang K (2020). Proteomics and metabolomics analysis reveal potential mechanism of extended-spectrum beta-lactamase production inEscherichia coli. Rsc Adv.

[34] Xu D, Xu Y, Ning N, Cui Q, Liu Z, Wang X, Liu D, Chen H, Kong MG (2018). Alteration of metabolite profiling by cold atmospheric plasma treatment in human myeloma cells. Cancer Cell Int.

[35] Teng  Z , Yu  Y , Zhu Z , Hong  SB , Yang  B, Zang  Y (2021). Melatonin elevated Sclerotinia sclerotiorum resistance via modulation of ATP and glucosinolate biosynthesis in Brassica rapa ssp. pekinensis. J Proteomics.

[36] Kind T, Wohlgemuth G, Lee DY, Lu Y, Palazoglu M, Shahbaz S, Fiehn O (2009). FiehnLib: Mass Spectral and Retention Index Libraries for Metabolomics Based on Quadrupole and Time-of-Flight Gas Chromatography/Mass Spectrometry. Anal Chem.

[37] Stefan A, Gentilucci L, Piaz FD, D'Alessio F, Santino F, Hochkoeppler A (2020). Purification from Deinococcus radiodurans of a 66 kDa ABC transporter acting on peptides containing at least 3 amino acids. Biochem Biophys Res Commun.

[38] Hao F, Tan Y, Lv X, Chen L, Yang F, Wang H, Du H, Wang L, Xu Y (2021). Microbial Community Succession and Its Environment Driving Factors During Initial Fermentation of Maotai-Flavor Baijiu. Front Microbiol.

