# Supplementary material for: Multi-omics analyses of the mechanism for the formation of soy sauce-like and soybean flavor in Bacillus subtilis BJ3-2
Source: BMC Microbiol. 2022 May 20;22:142. doi: 10.1186/s12866-022-02555-5 (PMC9121592; doi:10.1186/s12866-022-02555-5)
Supplement: Supplementary file 1 — Additional file 1: Fig. S1. Growth and physical characteristics. A: The color of fermentation broth; B: The OD value of fermentation broth; C: Gram staining of BJ3-2 cultured at 37°C; D: Gram staining of BJ3-2 cultured at 45°C; E: Gram staining of BJ3-2 cultured at 53°C. Fig. S2. Expression density distribution. Fig. S3. Data quality control of RNA-seq. A:Pearson correlation between smaples; B: Principal component analysis. Fig. S4. Analysis of DEGs. A: Volcano plot for group AT37 vs BT45; B: Volcano plot for group AT37 vs CT53; C:Volcano plot for group BT45 vs CT53; D: Cluster analysis of DEGs using H-cluster method. Fig. S5. Electrophoretograms of protein expressions. Fig. S6. Heat map analysis of DPGs in the AP37 vs BP45 A, AP37 vs CP53 B and BP45vs CP53 C. Fig. S6. Heat map analysis of DPGs in the AP37 vs BP45 A, AP37 vs CP53 B and BP45vs CP53 C. Fig. S7. Volcano plot analysis of DEPs in the AP37 vs BP45 A, AP37 vs CP53 B and BP45vs CP53C. Fig. S8.KEGG enrichment analysisfor group A37 vs B45 A, A37 vs C53 B and B45 vs C53 C. [file 12866_2022_2555_MOESM1_ESM.doc]

**Supplementary Figures**


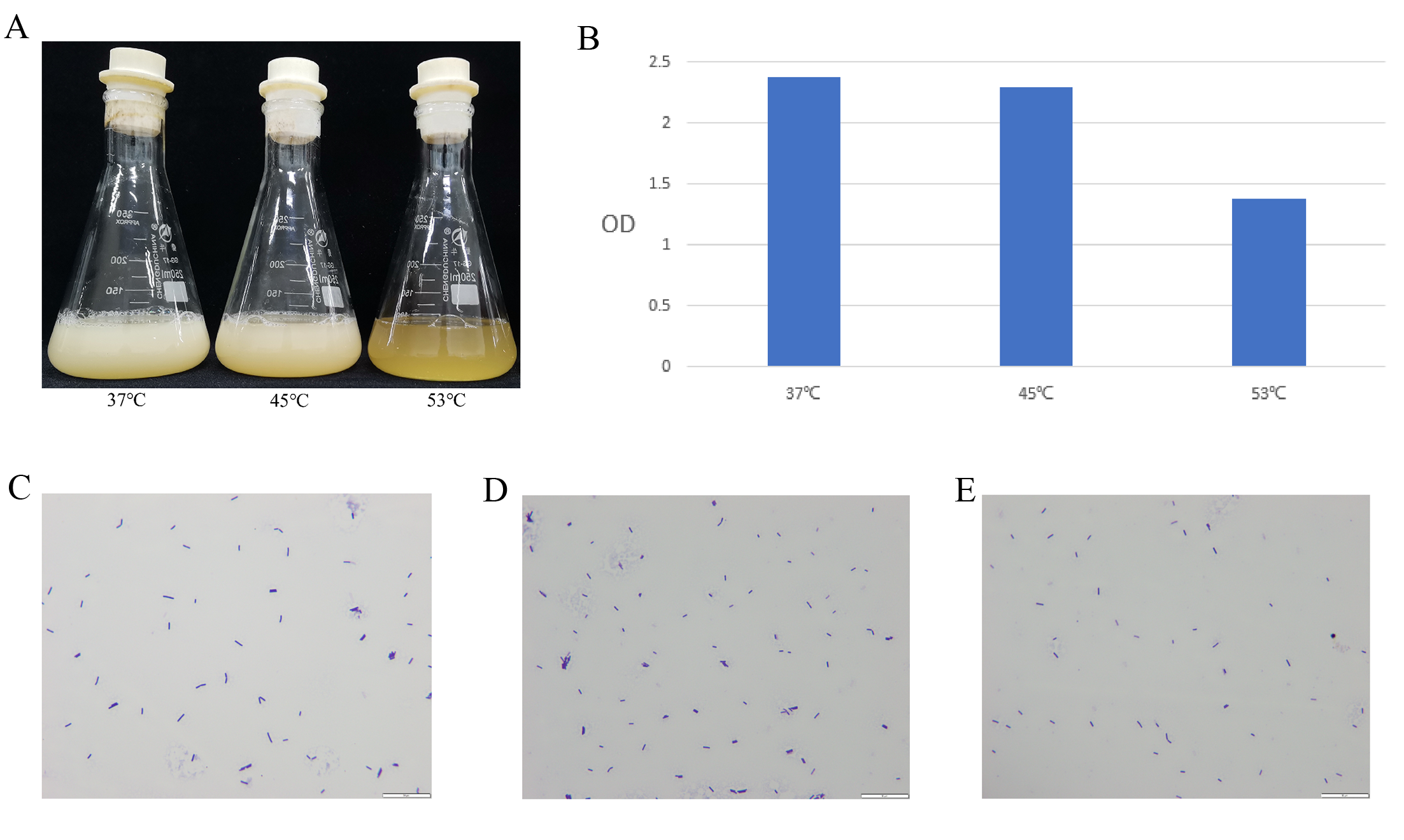


**Fig. S1**. Growth and physical characteristics. **A:** The color of fermentation broth; **B:** The OD value of fermentation broth; **C:** Gram staining of BJ3-2 cultured at 37°C; **D:** Gram staining of BJ3-2 cultured at 45°C; **E:** Gram staining of BJ3-2 cultured at 53°C*.*


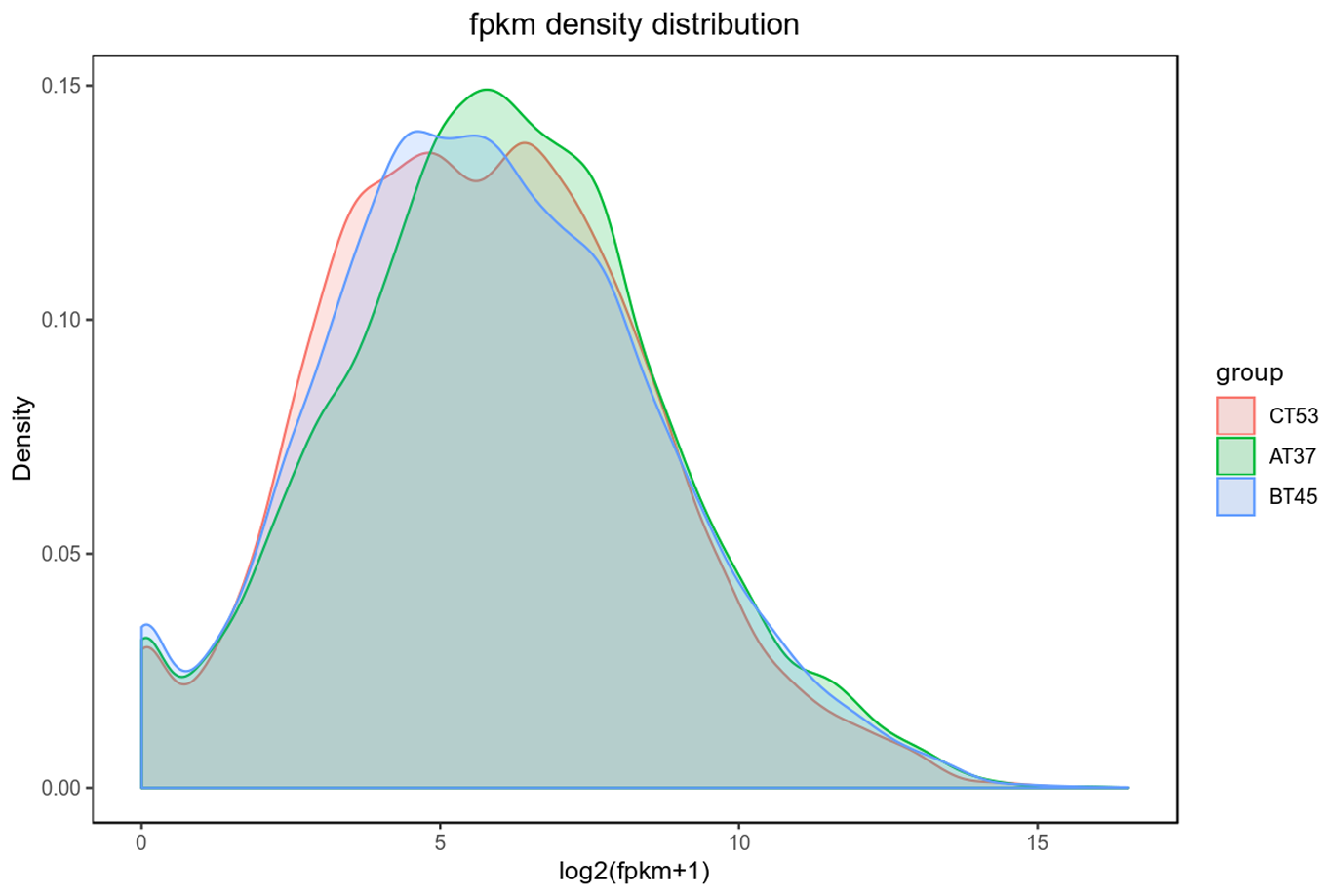


**Fig. S2**. Expression density distribution.


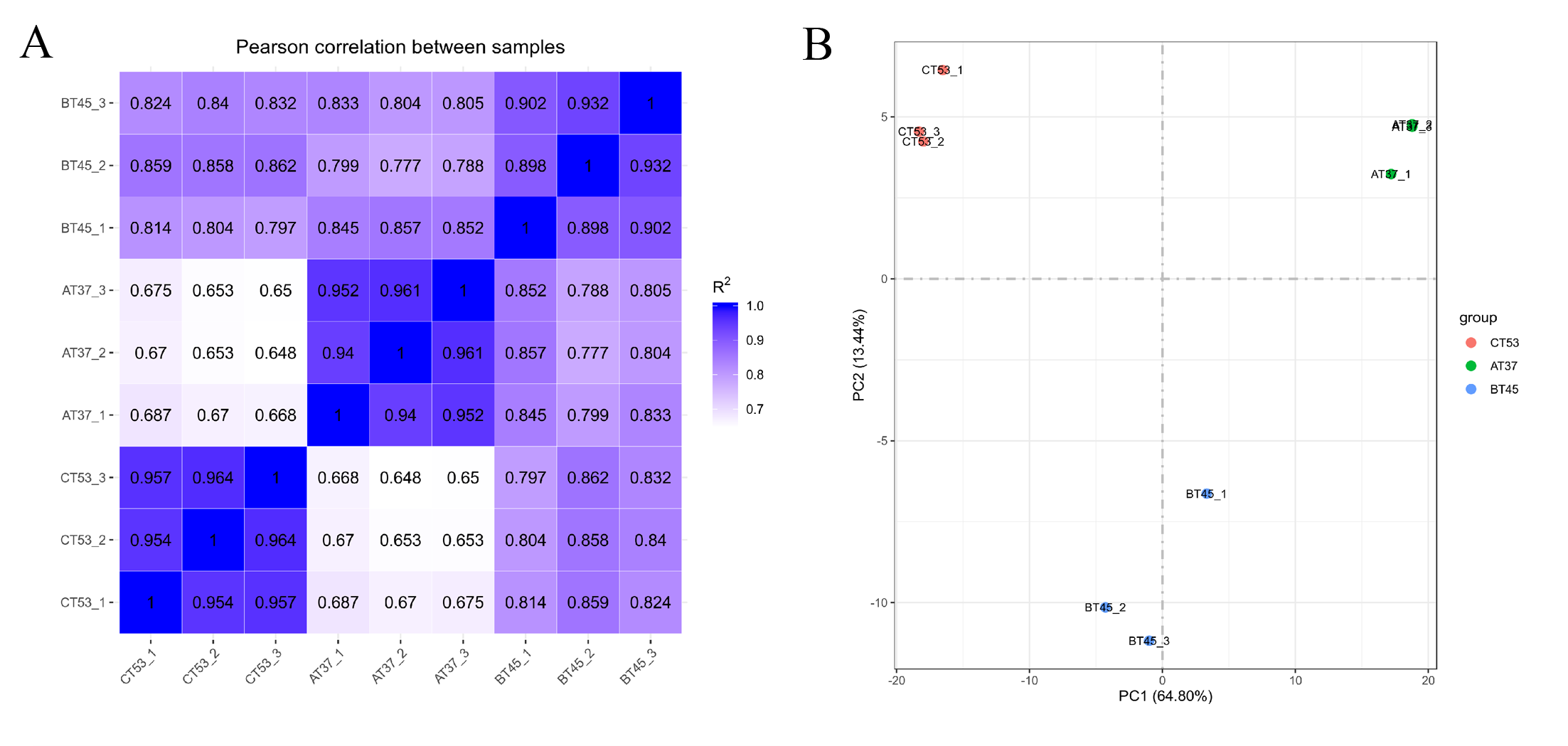


**Fig. S3**. Data quality control of RNA-seq. **A:**Pearson correlation between smaples; **B:** Principal component analysis.


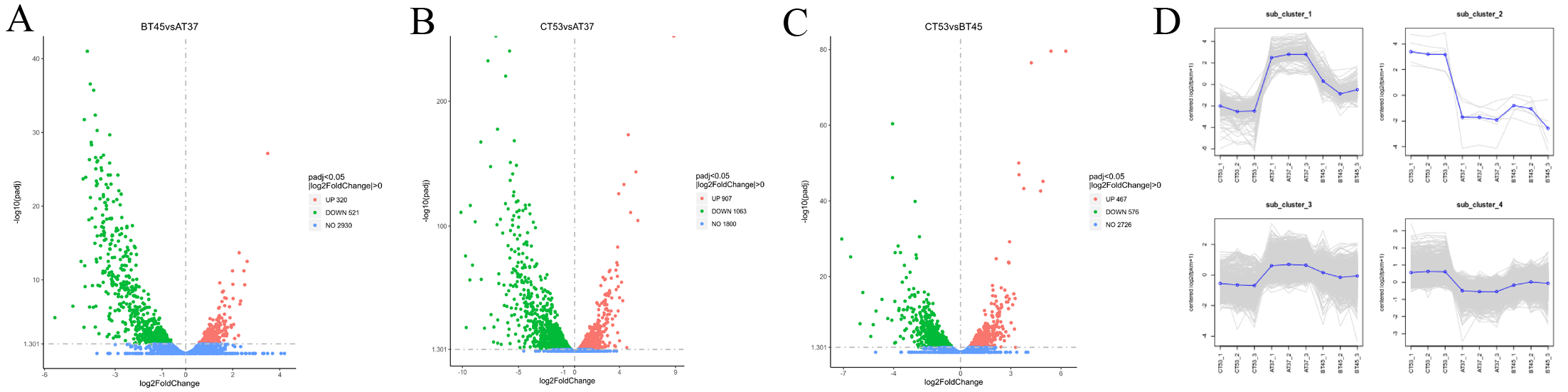


**Fig. S4**. Analysis of DEGs. **A:** Volcano plot for group AT37 vs BT45; **B:** Volcano plot for group AT37 vs CT53; **C:** Volcano plot for group BT45 vs CT53; **D:** Cluster analysis of DEGs using H-cluster method.


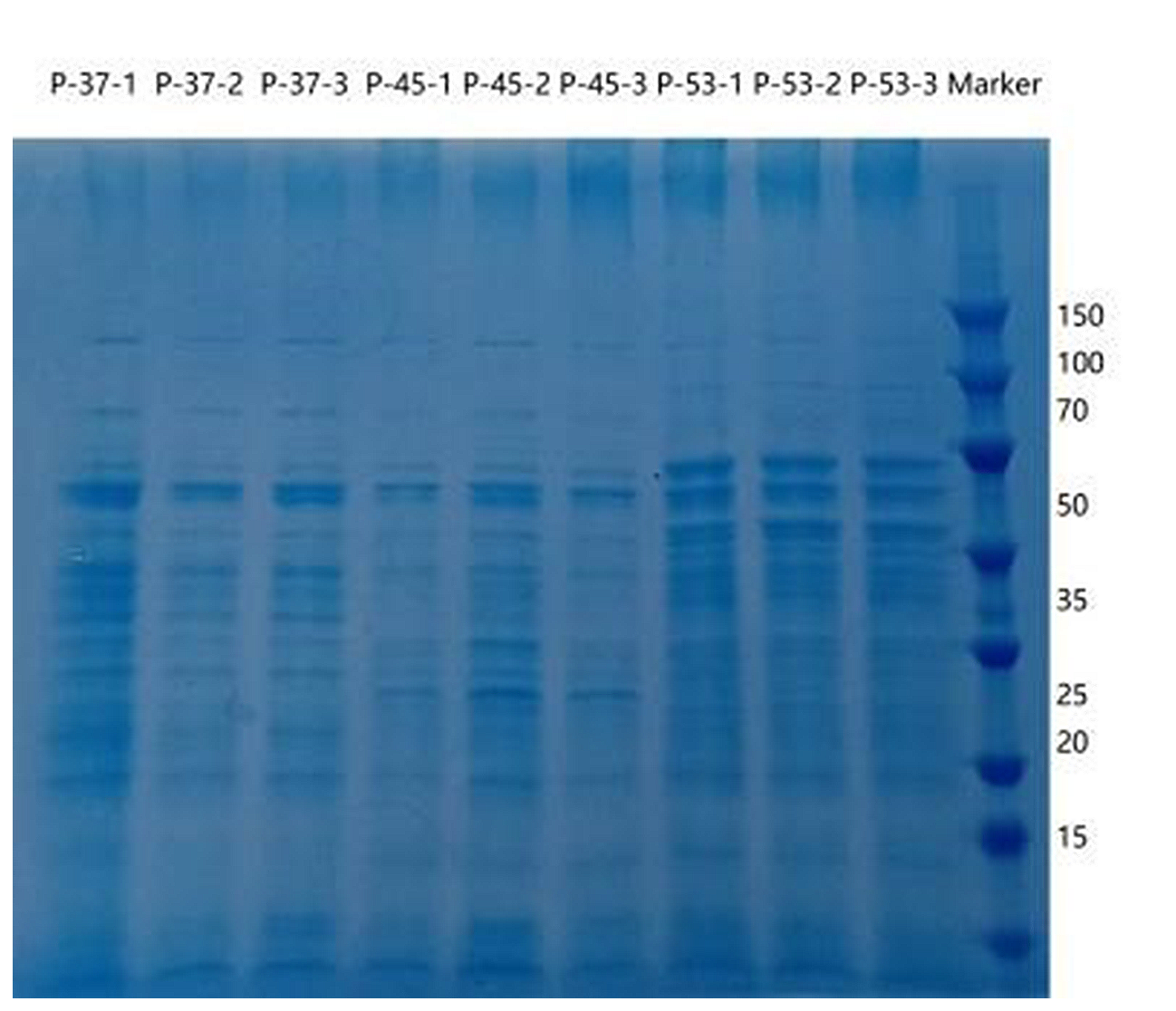


**Fig. S5**. Electrophoretograms of protein expressions.


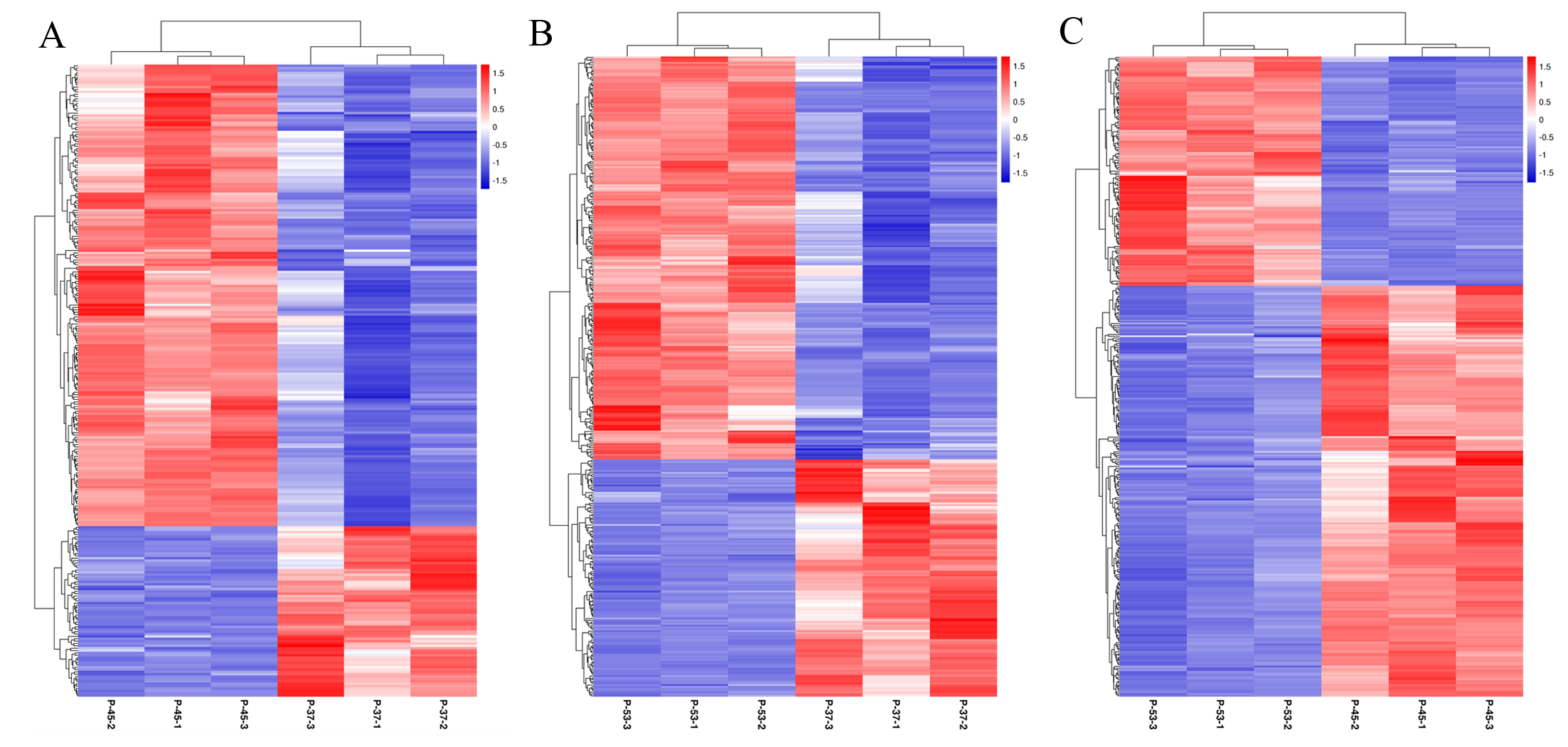


**Fig. S6**. Heat map analysis of DPGs in the AP37 vs BP45 (**A**), AP37 vs CP53 (**B**) and BP45vs CP53 (**C**).


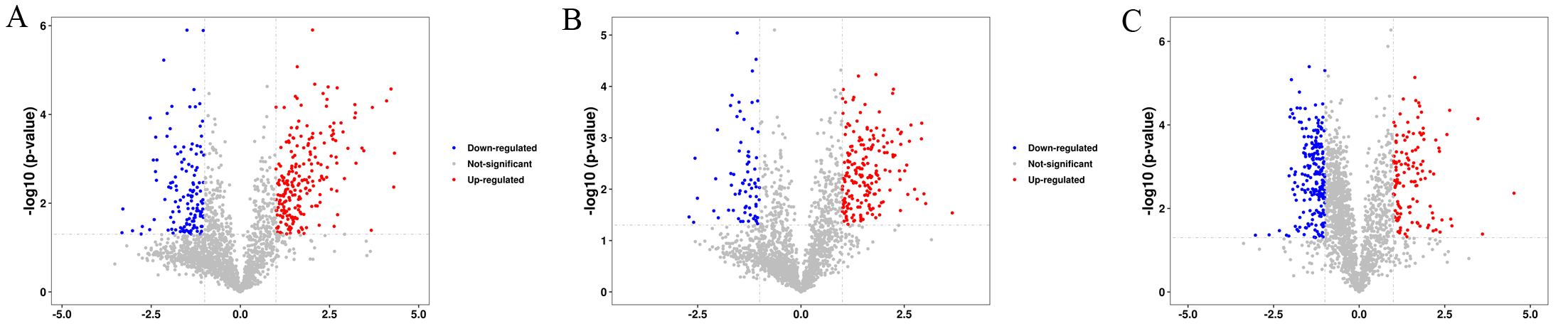


**Fig. S7**. Volcano plot analysis of DEPs in the AP37 vs BP45 (**A**), AP37 vs CP53 (**B**) and BP45vs CP53 (**C**).


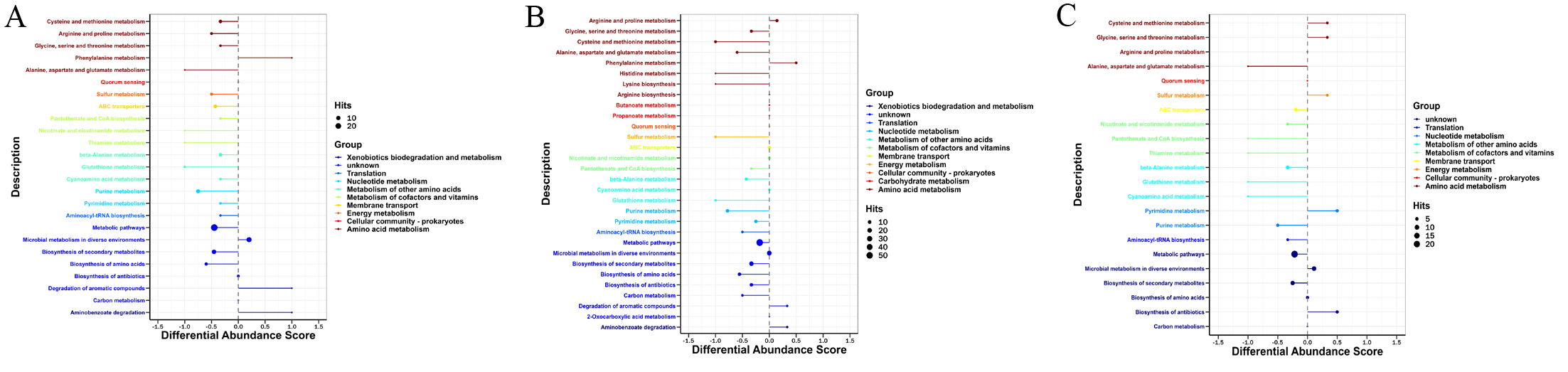


**Fig. S8**. KEGG enrichment analysis for group A37 vs B45(**A**), A37 vs C53 (**B**) and B45 vs C53(**C**).
